# Autoantigens in the trabecular meshwork and glaucoma‐specific alterations in the natural autoantibody repertoire

**DOI:** 10.1002/cti2.1101

**Published:** 2020-02-29

**Authors:** Vanessa M Beutgen, Carsten Schmelter, Norbert Pfeiffer, Franz H Grus

**Affiliations:** ^1^ Experimental and Translational Ophthalmology Department of Ophthalmology University Medical Center of the Johannes Gutenberg ‐ University Mainz Germany

**Keywords:** autoantigen, biomarker, glaucoma, immunoproteomics, natural autoantibodies, trabecular meshwork

## Abstract

**Objectives:**

Primary open‐angle glaucoma (POAG) is a neurodegenerative disorder leading to a gradual vision loss caused by progressive damage to the optic nerve. Immunological processes are proposed to be involved in POAG pathogenesis. Altered serological autoantibody levels have been frequently reported, but complete analyses of the natural autoantibodies with respect to disease‐related alterations are scarce. Here, we provide an explorative analysis of pathways and biological processes that may involve naturally immunogenic proteins and highlight POAG‐specific alterations.

**Methods:**

Mass spectrometry‐based antibody‐mediated identification of autoantigens (MS‐AMIDA) was carried out in healthy and glaucomatous trabecular meshwork (TM) cell lines, using antibody pools purified from serum samples of 30 POAG patients and 30 non‐glaucomatous subjects. Selected antigens were validated by protein microarray (*n* = 120). Bioinformatic assessment of identified autoantigens, including Gene Ontology (GO) enrichment analysis and protein–protein interaction networks, was applied.

**Results:**

Overall, we identified 106 potential autoantigens [false discovery rate (FDR) < 0.01], from which we considered 66 as physiological targets of natural autoantibodies. Twenty‐one autoantigens appeared to be related to POAG. Bioinformatic analysis revealed that the platelet‐derived growth factor receptor beta (PDGFRB) pathway involved in TM fibrosis was particularly rich in POAG‐related antigens. Antibodies to threonine‐tRNA ligase (TARS), component 1 Q subcomponent‐binding protein (C1QBP) and paraneoplastic antigen Ma2 (PNMA2) showed significantly (*P* < 0.05) higher levels in POAG patients as validated by protein microarray.

**Conclusion:**

This study provides new insights into autoimmunity in health and glaucoma. Bioinformatic analysis of POAG‐related autoantigens showed a strong association with the PDGFRB pathway and also increased levels of PNMA2, TARS, and C1QBP autoantibodies in the serum of POAG patients as potential glaucoma biomarkers.

## Graphical abstract

In this study, we identified 66 potential antigens in the trabecular meshwork (TM) as targets of natural autoimmunity. Of these antigens, 21 can be assigned a connection to primary open‐angle glaucoma (POAG). The platelet‐derived growth factor receptor beta (PDGFRB) pathway involved in TM fibrosis is especially represented by POAG‐related antigens.

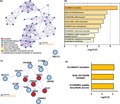

## Introduction

Primary open‐angle glaucoma (POAG) is an optic neuropathy generally characterised by an elevated intraocular pressure (IOP) above 21 mmHg,^1^ apoptosis of retinal ganglion cells and gradual loss of vision.^2^ In addition to old age,^3^ genetic predisposition^4^, ^5^ and decreased central corneal thickness,^6^ a high IOP is the major risk factor for POAG,^7^ but alone it is not sufficient for its onset. An elevated IOP in POAG is caused by an increased outflow resistance of the aqueous humour, triggered by pathological changes in the trabecular meshwork (TM). Outflow resistance in the TM is primarily caused by the cells and extracellular matrix of the juxtacanalicular region that is subject to constant remodelling to maintain aqueous humour efflux. In the glaucomatous eye, signalling pathways regulating extracellular matrix deposition, the actin cytoskeleton and cell–cell/cell–matrix connections are dysregulated.^8^, ^9^ Transforming growth factor β levels in aqueous humour are increased, leading to a rise in its downstream effector, connective tissue growth factor. This matricellular protein promotes the excessive accumulation of extracellular matrix proteins in the extracellular space, causing the TM tissue to be denser and thereby decreasing outflow.^10^

Furthermore, cross‐linking of the actin cytoskeleton in the TM increases. This limits the ability of the tissue to actively respond to changes in IOP by actomyosin‐mediated contraction of TM cells. The β‐integrin‐induced formation of cross‐linked actin networks increases the overall rigidity of the tissue and exacerbates the resistance in the TM outflow pathway.^11^ The stiffness of the tissue also accumulates with age,^12^ elevating the chances of drainage failure. The TM in glaucoma is about 20 times more rigid than in healthy tissue,^13^ reflecting enormous changes in TM biomechanics during the course of the disease. However, in addition, another form of open‐angle glaucoma without increased pathological IOP exists. This particular subgroup is known as normal‐tension glaucoma and comprises between 20% and 61% of all open‐angle glaucomas.^14^ This also suggests that other pathomechanisms are involved in the generation of glaucomatous damage to the optic nerve. In the case of normal‐tension glaucoma, a weakness of the lamina cribrosa has been proposed, making the retinal ganglion cells more prone to mechanical damage, even under physiological IOP.^15^ The optic nerve leaves the posterior eye through this mesh‐like tissue which has similar properties to the TM. Therefore, as the LC provides mechanical support to the optic nerve, compression and distortion of this structure may be involved in the damage to retinal ganglion cell axons in glaucoma.^16^, ^17^

Another factor that has been forwarded by previous studies is a possible involvement of immunological mechanisms. Several research groups already found altered levels of autoantibodies (AAbs) against diverse ocular antigens.^18^, ^19^, ^20^, ^21^, ^22^, ^23^ Interestingly, not only increased but also downregulated AAb reactivities have been identified,^24^, ^25^, ^26^ suggesting a complex alteration of the natural autoantibody repertoire. Many scientists have abandoned the paradigm of Paul Ehrlich's ‘horror autotoxicus’, which links AAbs only to pathological effects. The physiological immunome most likely fulfils more tasks than a direct defence against invading pathogens as part of humoral immunity. Instead, the immune system is also involved in homeostasis and clearance of cellular debris.^27^ The impairment of these functions can likely favor the development or progression of diseases.

Up to now, the human immunome has not been well explored. In this study, we looked at antigens in TM cells and their serological AAbs, as well as their function, to identify pathways involved in the emergence of autoantigens. Furthermore, we searched for hints of any altered immunogenicity of these proteins in POAG and validated an increase in AAbs against of paraneoplastic antigen Ma2 (PNMA2), threonine‐tRNA ligase (TARS) and complement component 1 Q subcomponent‐binding protein (C1QBP) by antigen microarray.

## Results

### Identification of autoantigens in human TM cells using MS‐AMIDA

Mass spectrometry‐based antibody‐mediated identification of autoantigens (MS‐AMIDA) was used to detect serological AAbs to antigens in human TM cells. The workflow comprises the isolation of IgGs from human non‐glaucomatous serum (for characteristics, see Table 1, MS‐AMIDA CTRL group) and their cross‐linking to protein G‐coated magnetic beads. These beads were subsequently incubated with TM cell lysates from immortalised human cell lines. The putative autoantigens bind to the immobilised antibodies, and after on‐bead tryptic digestion, they were identified by high‐resolution MS. The IgG repertoire of non‐glaucomatous subjects against antigens from healthy (HTM) and glaucomatous TM (GTM) cells was tested. Also, negative controls, without IgG, and a mock control were included, which was incubated only with lysis buffer instead of cell lysate. An overview of the experimental design is depicted in Table 2.

**Table 1:** Characteristics of the study population

	POAG	CTRL
MS‐AMIDA		
Samples (*n*)	30 (3 pools)	30 (3 pools)
Sex (m/f)	17/13	15/15
Mean age (±SD)	63.93 ± 9.13	64.5 ± 9.82
Eye surgery	None	None
Autoimmune disease	No reported	No reported
Other eye disease	Cataract (9 of 30)	Cataract (7 of 30)
Microarray validation		
Samples (*n*)	120	120
Sex (m/f)	65/55	65/55
Mean age (±SD)	67.25 ± 10.95	67.17 ± 11.89

**Table 2:** Experimental design for the initial MS‐AMIDA profiling

ID	Sample name	IgG source	Protein source	Comment
NC1	Negative control 1	None	Healthy TM cells	Unspecific binding to beads
NC2	Negative control 2	None	Glaucomatous TM cells	Unspecific binding to beads
MA	Mock A	POAG	Lysis buffer	Mock control; 3 replicates
MB	Mock B	CTRL	Lysis buffer	Mock control; 3 replicates
PH	POAG/HTM	POAG	Healthy TM cells	3 replicates
PG	POAG/GTM	POAG	Glaucomatous TM cells	3 replicates
CH	CTRL/HTM	CTRL	Healthy TM cells	3 replicates
CG	CTRL/GTM	CTRL	Glaucomatous TM cells	3 replicates

**Figure 1:**
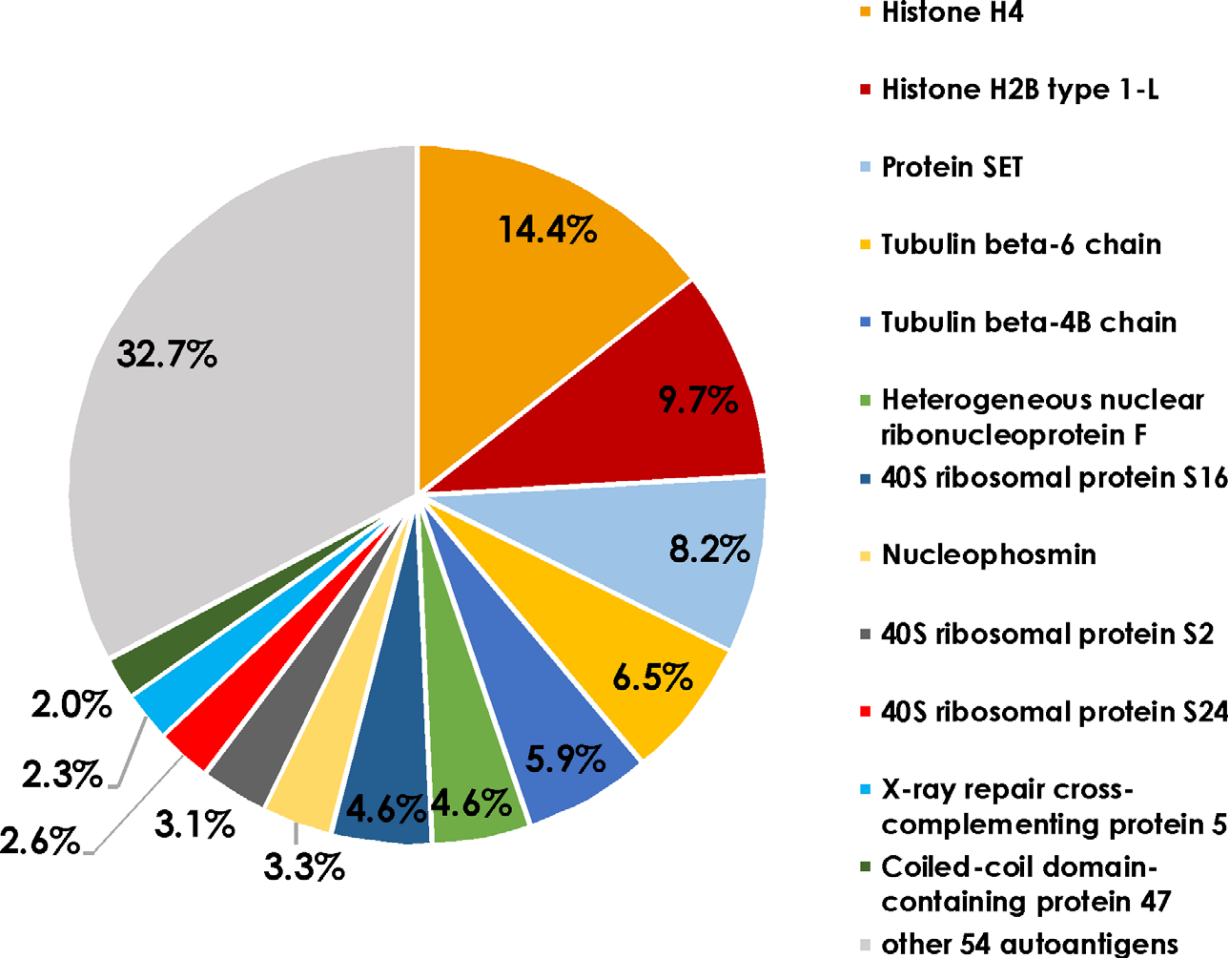
iBAQ values of captured autoantigens using CTRL serum and HTM cells. The iBAQ values are proportional to the molar quantity of the respective antigen measured by mass spectrometry. These proportions represent the abundance of the associated autoantibodies in natural autoimmunity. The diagram shows the distribution of different autoantigens from healthy TM cells captured by antibodies in the control sera (*n* = 3). The percentage of total peptide intensities is shown.

With this approach, we identified 157 proteins in all groups, which were reproducibly found in all three replicates of at least one experimental group, with high confidence [false discovery rate (FDR) < 0.01]. Fifty‐one of these proteins were also found in the negative control and mock control groups and were excluded from further analysis. We considered the remaining 106 proteins as potential autoantigens (Supplementary table 1). To elucidate the natural autoantibody repertoire detectable with this method, the control experimental group, incubated with HTM cell lysates (‘CH’), was evaluated with special interest. In this group, 66 antigens were identified (Table 3). The analysis of the intensity‐based absolute quantification (iBAQ) values revealed that 12 antigens contribute to 67.3% of all identified proteins (Figure 1). The most abundant antigens were histone H4 with 14.4% and histone H2B type 1‐L (9.7%), followed by the histone H4‐binding partner protein SET with 8.2%. Other antigens among the top 10 most abundant were proteins from the tubulin beta chain (TBB6 6.5% and TBB4B 5.9%) and 40S ribosomal proteins (RS16 4.6%, RS2 3.3%, RS24 2.6%). Forty of the (auto)‐antibody‐captured proteins showed relative low abundance with < 1%. A complete list with all iBAQ values can be found in Table 3.

**Table 3:** Autoantigens identified in HTM cells, captured by autoantibodies of CTRL sera (Group ID: ‘CH’)

No.	Entry name (*_HUMAN)	UniProt accession	Protein name	Sequence coverage (%)	No. of peptides	No. of unique peptides	iBAQ (%)
1	FLNA	P21333	Filamin‐A	11	20	16	1.24
2	TBB6	Q9BUF5	Tubulin beta‐6 chain	46.6	15	6	6.51
3	SYEP	P07814	Bifunctional glutamate/proline‐tRNA ligase	11	12	12	1.15
4	SET	Q01105	Protein SET	19.3	3	3	8.23
5	TBB4B	P68371	Tubulin beta‐4B chain	51.7	17	1	5.88
6	SYIC	P41252	Isoleucine‐tRNA ligase, cytoplasmic	12.1	12	12	0.99
7	FAS	P49327	Fatty acid synthase	4.8	7	7	0.44
8	TERA	P55072	Transitional endoplasmic reticulum ATPase	12.8	6	6	1.14
9	H4	P62805	Histone H4	35.9	4	4	14.39
10	XRCC5	P13010	X‐ray repair cross‐complementing protein 5	23.8	8	8	2.28
11	RS2	P15880	40S ribosomal protein S2	18.8	4	4	3.09
12	H2B1L	Q99880	Histone H2B type 1‐L	19	2	2	9.69
13	HNRPF	P52597	Heterogeneous nuclear ribonucleoprotein F	11.3	3	2	4.65
14	RS16	P62249	40S ribosomal protein S16	14.4	2	2	4.6
15	SYTC	P26639	Threonine‐tRNA ligase, cytoplasmic	11.1	7	7	1.77
16	PLPHP	O94903	Pyridoxal phosphate homeostasis protein	17.5	3	3	1.81
17	HS90B	P08238	Heat shock protein HSP 90‐beta	20.9	10	7	1.41
18	ATPA	P25705	ATP synthase subunit alpha, mitochondrial	15.9	5	5	1.56
19	SERPH	P50454	Serpin H1	14.1	4	4	1.6
20	NPM	P06748	Nucleophosmin	12.6	3	3	3.29
21	MCM7	P33993	DNA replication licensing factor MCM7	7.4	4	4	0.55
22	CCD47	Q96A33	Coiled‐coil domain‐containing protein 47	14.3	4	4	2.03
23	ADT2	P05141	ADP/ATP translocase 2	14.4	4	2	1.69
24	SYRC	P54136	Arginine‐tRNA ligase, cytoplasmic	17.7	10	10	0.74
25	IF4A1	P60842	Eukaryotic initiation factor 4A‐I	27.8	6	6	1.69
26	IQGA1	P46940	Ras GTPase‐activating‐like protein IQGAP1	2.8	2	2	0.1
27	HNRPK	P61978	Heterogeneous nuclear ribonucleoprotein K	7.8	3	3	1.01
28	EIF3L	Q9Y262	Eukaryotic translation initiation factor 3 subunit L	3	1	1	0.54
29	SYK	Q15046	Lysine‐tRNA ligase	8.9	4	4	0.5
30	SYDC	P14868	Aspartate‐tRNA ligase, cytoplasmic	7.8	4	4	0.66
31	SRP68	Q9UHB9	Signal recognition particle subunit SRP68	12.1	5	5	0.51
32	TBA1A	Q71U36	Tubulin alpha‐1A chain	46.1	17	1	1.07
33	RS24	P62847	40S ribosomal protein S24	9	1	1	2.63
34	RL15	P61313	60S ribosomal protein L15	5.9	1	1	1.3
35	EIF3E	P60228	Eukaryotic translation initiation factor 3 subunit E	2.9	1	1	0.45
36	EIF3F	O00303	Eukaryotic translation initiation factor 3 subunit F	5.3	1	1	0.76
37	SYQ	P47897	Glutamine‐tRNA ligase	9	5	5	0.36
38	TCPZ	P40227	T‐complex protein 1 subunit zeta	7.2	2	2	0.47
39	MYO1C	O00159	Unconventional myosin‐Ic	1.2	1	1	0.05
40	EIF3A	Q14152	Eukaryotic translation initiation factor 3 subunit A	3.5	4	4	0.16
41	DEST	P60981	Destrin	10.3	1	1	1.63
42	EIF3B	P55884	Eukaryotic translation initiation factor 3 subunit B	3.4	1	1	0.26
43	SYMC	P56192	Methionine‐tRNA ligase, cytoplasmic	1.2	1	1	0.33
44	NSUN2	Q08J23	tRNA (cytosine(34)‐C(5))‐methyltransferase	3.9	2	2	0.43
45	COPG1	Q9Y678	Coatomer subunit gamma‐1	4.9	2	2	0.2
46	MCM3	P25205	DNA replication licensing factor MCM3	2.7	2	2	0.13
47	PSMD2	Q13200	26S proteasome non‐ATPase regulatory subunit 2	4.5	2	2	0.12
48	OLA1	Q9NTK5	Obg‐like ATPase 1	3.8	1	1	0.19
49	RS5	P46782	40S ribosomal protein S5	13.7	1	1	0.9
50	ACTN1	P12814	Alpha‐actinin‐1	3.8	3	3	0.27
51	PYRG1	P17812	CTP synthase 1	2.7	1	1	0.19
52	PPIL4	Q8WUA2	Peptidyl‐prolyl cis‐trans isomerase‐like 4	8.7	2	2	0.34
53	NU160	Q12769	Nuclear pore complex protein Nup160	1.3	1	1	0.06
54	P5CS	P54886	Delta‐1‐pyrroline‐5‐carboxylate synthase	4.5	2	2	0.14
55	TKT	P29401	Transketolase	6.9	2	2	0.21
56	DCTN1	Q14203	Dynactin subunit 1	3.7	3	3	0.08
57	CLH1	Q00610	Clathrin heavy chain 1	0.5	1	1	0.07
58	PRP8	Q6P2Q9	Pre‐mRNA‐processing‐splicing factor 8	0.9	1	1	0.03
59	DX39A	O00148	ATP‐dependent RNA helicase DDX39A	4.9	2	2	0.31
60	TIM50	Q3ZCQ8	Mitochondrial import inner membrane translocase subunit TIM50	2.8	1	1	0.4
61	CPSF7	Q8N684	Cleavage and polyadenylation specificity factor subunit 7	5.3	1	1	0.25
62	SPT5H	O00267	Transcription elongation factor SPT5	1.7	1	1	0.07
63	IMB1	Q14974	Importin subunit beta‐1	1	1	1	0.06
64	RT35	P82673	28S ribosomal protein S35, mitochondrial	3.1	1	1	0.17
65	TGFI1	O43294	Transforming growth factor beta‐1‐induced transcript 1 protein	4.3	1	1	0.11
66	DDX46	Q7L014	Probable ATP‐dependent RNA helicase DDX46	1	1	1	0.05

Proteins were reproducibly identified in all three replicates. MaxQuant iBAQ values were used to calculate protein abundance.

### Characterisation of natural autoantigens by GO enrichment and pathway analysis

**Figure 2:**
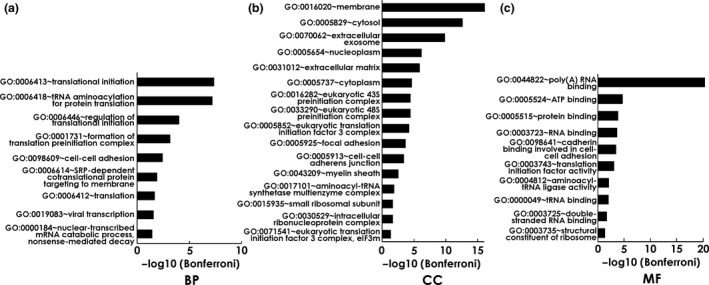
GO enrichment analysis of autoantigens from HTM captured by autoantibodies in CTRL sera (*n* = 3), representing natural autoimmunity. Negative log_10_‐transformed *P*‐values of significantly enriched GO terms (*P* < 0.05 with Bonferroni correction) are shown in **(a)** biological process (BP), **(b)** cellular component (CC) and **(c)** molecular function (MF).

Gene Ontology (GO) enrichment was analysed using DAVID (https://david.ncifcrf.gov/) to get an overview of the characteristics of the identified targets of the natural AAbs in the CH experiment group. Figure 2 shows significantly enriched GO terms among the autoantigens for biological process (A), cellular component (B) and molecular function (C).

The analysis showed that the antigens are profoundly involved in several processes associated with translation‐ and RNA‐related processes. Furthermore, the detected immunogenic proteins are involved in virus‐associated processes, as well as cell–cell adhesion. Highlighting the molecular functions of the autoantigens, nearly all of them showed the ability to bind poly (A) or other forms of RNA. Also, the binding of cadherin in cell–cell adhesion represented a frequent function among the autoantigens captured by antibodies from the control sera. We also found marked enrichment of the immunogenic proteins in the cellular components membrane, cytosol and extracellular exosomes.

### Identification of POAG‐related autoantigens

**Figure 3:**
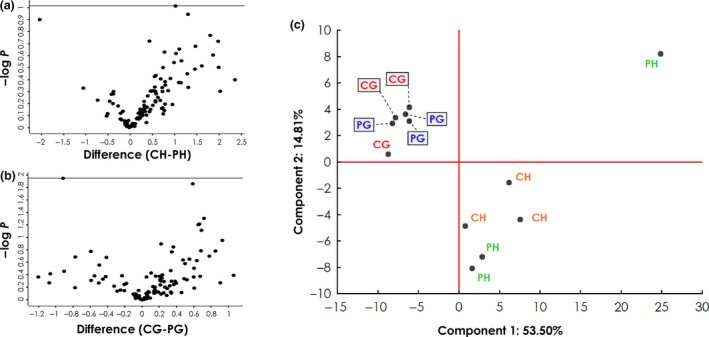
Analysis of autoantigen abundance in different experiment groups (IDs: PH, CH, PG, CG). Detailed information about the experimental groups can be found in Table 2. **(a, b)** Volcano plots showing negative log_10_‐transformed *P*‐values (*t*‐test with permutation‐based FDR to correct for multiple testing; *y*‐axis) against the differences of the means (log_2_; *x*‐axis) of two groups. **(a)** Comparison of antigens from healthy TM cell lysates captured by antibodies from control (CH; *n* = 3) and POAG sera (PH; *n* = 3). **(b)** Comparison of antigens from glaucomatous TM cell lysates captured by antibodies from control (CG; *n* = 3) and POAG sera (PG; *n* = 3). **(c)** Principal component analysis, including all four experiment groups, reveals that most of the variance between the groups can be attributed to the cell line used rather than the antibody source.

**Figure 4:**
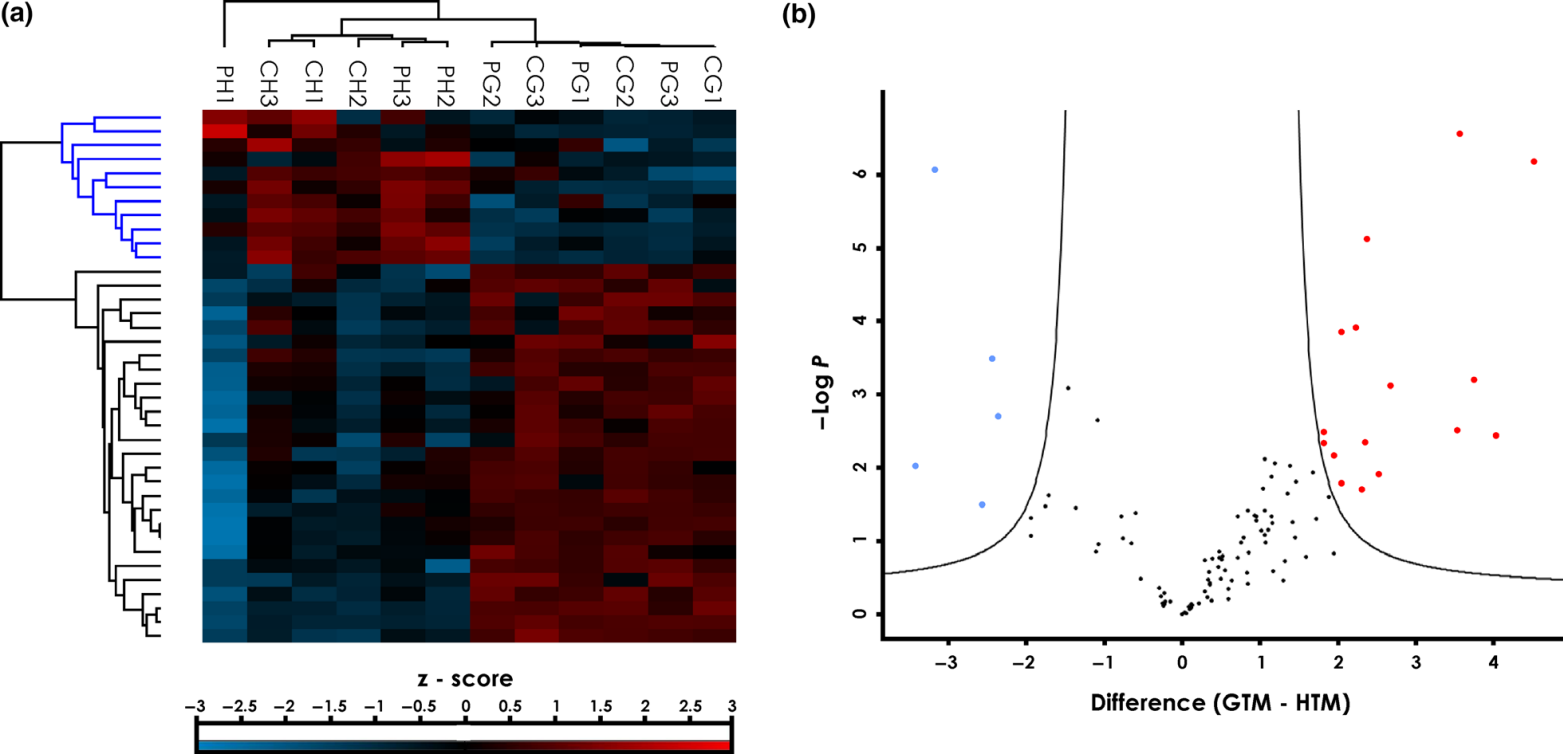
Statistical analysis of captured autoantigens from HTM and GTM cells. **(a)** Hierarchical clustering of antigens in single samples with significantly different abundance between the cell lines HTM and GTM (38 proteins; *t*‐test with permutation‐based FDR < 0.01). Clustering is based on *z*‐score‐transformed LFQ intensities. **(b)** Volcano plot for candidate selection. Highlighted dots represent proteins with significant differences between cell lines and a log_2_ fold change of 2 or more, to ensure relevant effect size. Blue dots represent antigens with lower levels and red dots antigens with higher levels in the GTM samples than HTM samples.

The involvement of immunological processes has been frequently shown for several neurodegenerative diseases and for the ocular neuropathy, POAG. To identify antigens that are related to POAG, we analysed autoantibody abundance in the sera of POAG patients and a non‐glaucomatous control group (Table 1). To this end, the proteins identified by MS‐AMIDA were quantified using MaxQuant label‐free quantification (LFQ) intensities. The experimental groups CH and PH (Figure 3a) and also CG and PG (Figure 3b) were analysed for significant differences in autoantibody levels. These were tested using Student's *t*‐test with permutation‐based FDR (< 0.01) to adjust for multiple testing. The test, however, revealed no significant alterations. Principal component analysis revealed that there is a higher association of the autoantibody levels with the TM cell line used than with the antibody source (Figure 3c). Component 1 and Component 2 explain 68.11% of the variance in the data (see also scree plot in Supplementary figure 1). The influence of single autoantibody levels on the components can be evaluated by observing the component coefficients in Supplementary table 2. Component 1 is mainly influenced by EIF5A1, H4, C1QBP and PNMA2, whereas Component 2 is mainly influenced by NUCL, NSUN2, HS90B and ACTN1. Hierarchical clustering of these antigens based on Euclidean distances (Figure 4a) supports the findings from the principal component analysis (Figure 3c). The experimental groups cluster together based on the specific TM cells, rather than the serum source. The antigens showed two clusters with 11 antigens having lower levels in the glaucomatous TM cells and 27 with increased levels. Therefore, we also examined the differences in the captured autoantigens between GTM and HTM cells. Here, 38 (auto)‐antibody‐captured proteins were found at significantly different levels (Supplementary table 3). We wanted to use a larger sample size to analyse whether different autoantibody serum levels caused these observed changes. To select suitable candidates for further validation, antigens were not only analysed for significant hits but also their fold change (Figure 4b). Only antigens with an FDR < 0.01 and a log_2_ fold change of > 2 were considered eligible. A total of 21 autoantigens passed these criteria (Table 4). From the subset of the 21 eligible autoantigens, nine targets were chosen for further validation by a protein microarray analysis.

**Table 4:** Candidate biomarkers with *P*‐value < 0.05 and log_2_ fold change of > 2 (log_2_ differences of the means)

UniProt accession	Entry name	Gene	Protein	−Log(*P*‐value)	Difference (GTM – HTM)
P62805	H4_HUMAN	HIST1H4A	Histone H4	2.028	−3.428
Q08J23	NSUN2_HUMAN	NSUN2	tRNA (cytosine(34)‐C(5))‐methyltransferase	6.063	−3.172
P13010	XRCC5_HUMAN	XRCC5	X‐ray repair cross‐complementing protein 5	1.496	−2.564
P12814	ACTN1_HUMAN	ACTN1	Alpha‐actinin‐1	3.484	−2.437
P26639	SYTC_HUMAN	TARS	Threonine‐tRNA ligase, cytoplasmic	2.706	−2.362
Q6UVK1	CSPG4_HUMAN	CSPG4	Chondroitin sulphate proteoglycan 4	2.490	1.816
Q02878	RL6_HUMAN	RPL6	60S ribosomal protein L6	2.340	1.819
Q14566	MCM6_HUMAN	MCM6	DNA replication licensing factor MCM6	2.164	1.950
Q96AB3	ISOC2_HUMAN	ISOC2	Isochorismatase domain‐containing protein 2	3.856	2.043
Q9NTK5	OLA1_HUMAN	OLA1	Obg‐like ATPase 1	1.786	2.053
Q00005	2ABB_HUMAN	PPP2R2B	Serine/threonine‐protein phosphatase 2A 55 kDa regulatory subunit B beta isoform	3.909	2.226
P10809	CH60_HUMAN	HSPD1	60 kDa heat shock protein, mitochondrial	1.705	2.305
P42704	LPPRC_HUMAN	LRPPRC	Leucine‐rich PPR motif‐containing protein, mitochondrial	2.342	2.350
O00159	MYO1C_HUMAN	MYO1C	Unconventional myosin‐Ic	5.121	2.368
P63104	1433Z_HUMAN	YWHAZ	14‐3‐3 protein zeta/delta	1.907	2.527
P46940	IQGA1_HUMAN	IQGAP1	Ras GTPase‐activating‐like protein IQGAP1	3.117	2.680
Q07021	C1QBP_HUMAN	C1QBP	Complement component 1 Q subcomponent‐binding protein, mitochondrial	2.513	3.536
Q9ULC4	MCTS1_HUMAN	MCTS1	Malignant T‐cell‐amplified sequence 1	6.556	3.560
P27708	PYR1_HUMAN	CAD	CAD protein	3.204	3.754
P63241	IF5A1_HUMAN	EIF5A	Eukaryotic translation initiation factor 5A‐1	2.437	4.028
Q9UL42	PNMA2_HUMAN	PNMA2	Paraneoplastic antigen Ma2	6.185	4.521

### Characterisation of POAG‐related autoantigens

**Figure 5:**
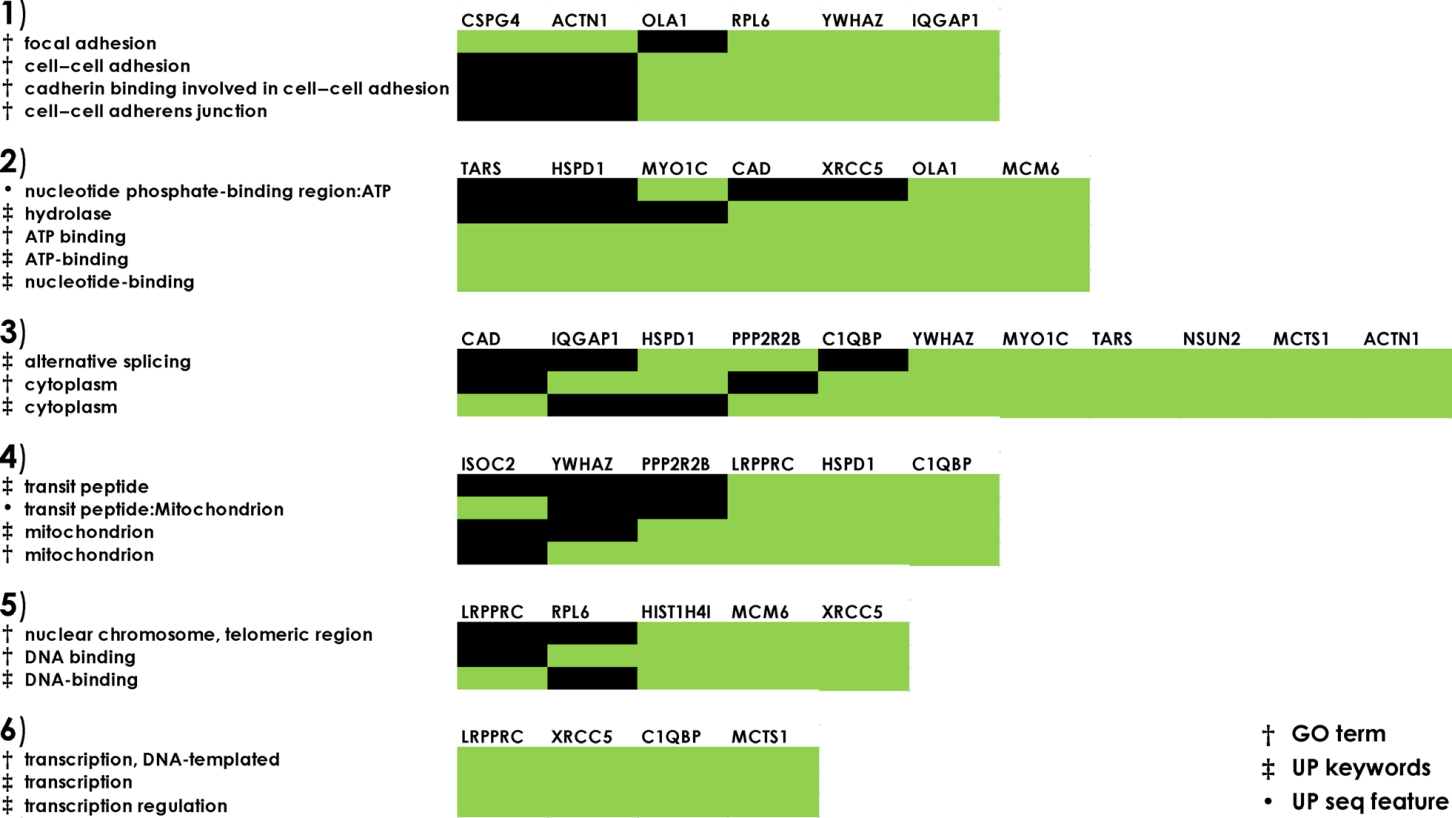
DAVID functional annotation clusters of potentially POAG‐related autoantigens. Nineteen out of the 21 included proteins showed an enrichment in at least one cluster. Enrichment scores: cluster 1 (2.54), cluster 2 (1.7), cluster 3 (1.59), cluster 4 (1.32), cluster 5 (1.02) and cluster 6 (0.39). The analysis includes Gene Ontology terms (GO terms), UniProt keywords (UP keywords) and UniProt sequence features (UP seq feature). Green indicates enrichment in the cluster for the respective protein.

**Figure 6:**
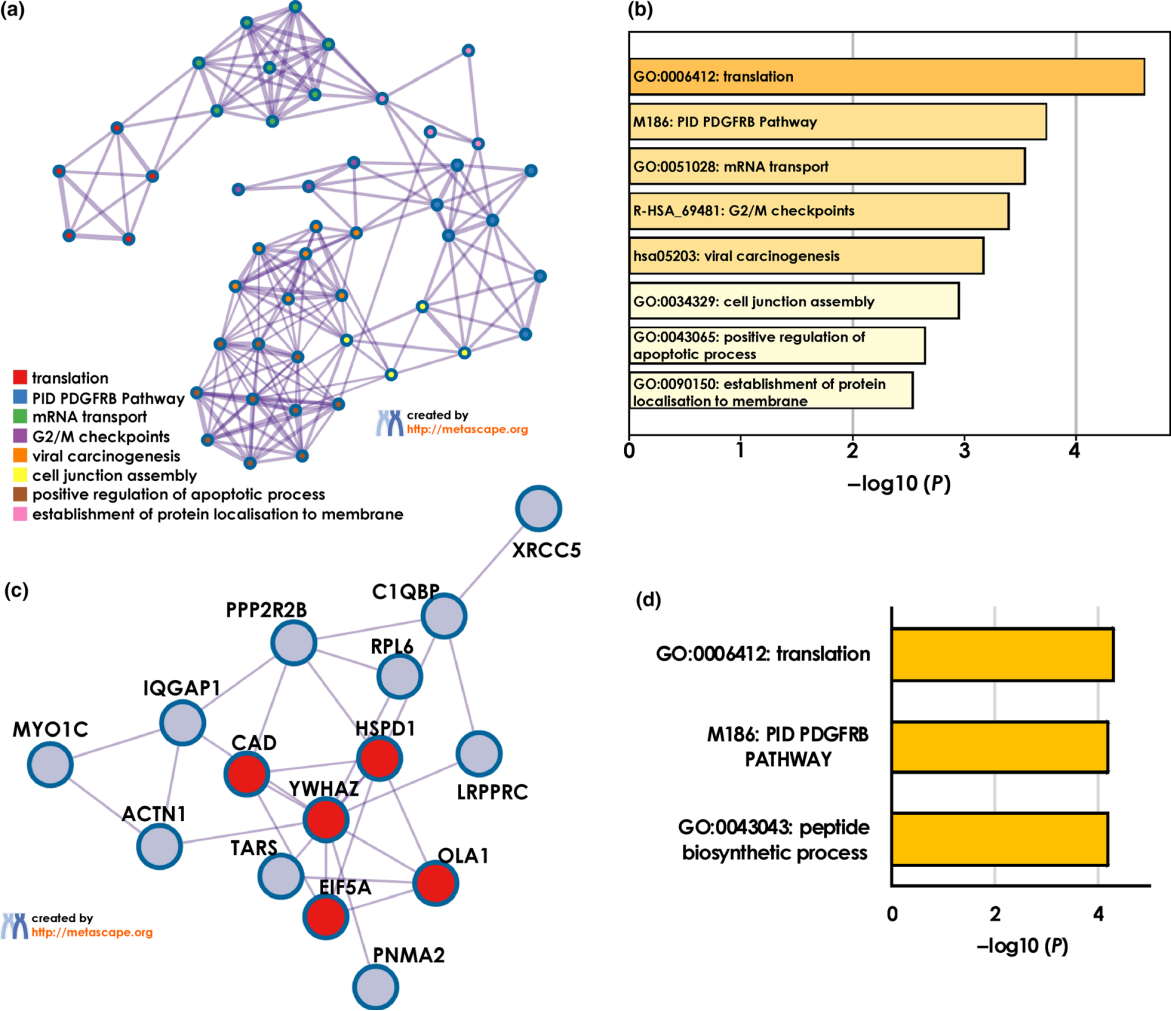
Metascape analysis of POAG‐related autoantigens. **(a)** Network of enriched GO and KEGG terms coloured by cluster. **(b)** Enriched GO and KEGG terms from **(a)** with corresponding *P*‐values. **(c)** Protein–protein interaction network with densely connected components (red) identified by the MCODE algorithm. **(d)** Enrichment analysis for MCODE cluster only.

To investigate in which biological processes these possibly POAG‐related autoantigens are involved, we used DAVID's functional annotation clusters and also Metascape enrichment and pathway analysis. DAVID identified six functional clusters among 19 of the 21 targets analysed using GO terms, UniProt (UP) keywords and UP sequence features (seq feature) (Figure 5). These clusters comprise cell–cell adhesion and junctions (cluster 1), nucleotide and ATP‐binding (cluster 2), cytoplasmic proteins with different isoforms (cluster 3), mitochondria‐associated proteins (cluster 4), DNA‐binding proteins (cluster 5) and transcription (cluster 6). The analysis with Metascape revealed eight enriched GO and Kyoto Encyclopaedia of Genes and Genomes (KEGG) terms (Figure 6a and b). The target antigens were enriched in translation, mRNA transport, cell junction assembly, positive regulation of apoptotic process and establishment of protein localisation to membrane, as well as viral carcinogenesis pathways, G2/M checkpoints and the platelet‐derived growth factor receptor beta (PDGFRB) pathway. The protein–protein interaction (PPI) network shows only antigens that are known to interact with at least one other of the identified targets (Figure 6c). This analysis showed that 15 of the 21 targets are connected in the PPI network. Of these, the Metascape molecular complex detection (MCODE) algorithm identified one densely connected network component, consisting of the proteins CAD, HSPD1, YWHAZ, OLA1 and EIF5A. The independent enrichment analysis for this MCODE component revealed an enrichment of the five antigens in translation, peptide biosynthetic process and the PDGFRB pathway (Figure 6d).

### Validation of selected targets by protein microarray

**Figure 7:**
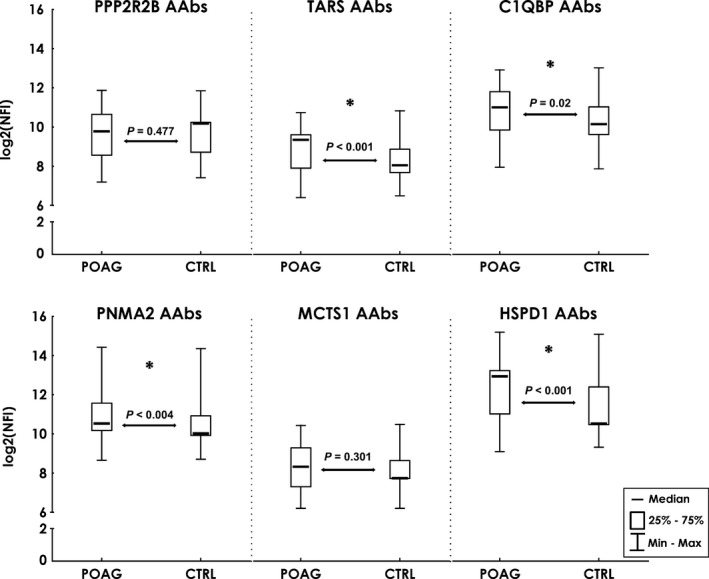
Results of the microarray validation. Comparison of autoantibody levels in POAG (*n* = 120) and CTRL (*n* = 120) serum. Median log_2_‐transformed normalised fluorescence intensities. Median intensities with 25–75% percentiles (box) and min/max values (whiskers). *P*‐values from a Mann–Whitney *U*‐test. Asterisks mark significant differences at *P* < 0.05.

We chose alpha‐actinin‐1 (ACTN1), serine/threonine‐protein phosphatase 2A 55 kDa regulatory subunit B beta isoform (PPP2R2B), mitochondrial complement component 1 Q subcomponent‐binding protein (C1QBP), malignant T‐cell‐amplified sequence 1 (MCTS1), paraneoplastic antigen Ma2 (PNMA2), Ras GTPase‐activating‐like protein IQGAP1 (IQGAP1), unconventional myosin‐1c (MYO1C) and cytoplasmic threonine‐tRNA ligase (TARS) to be analysed by protein microarray. Even though it is not a new target, heat shock protein 60 (HSP60) has been included as a well‐defined glaucoma‐related antigen to serve as a positive control. The targets were obtained as recombinant proteins and were used to prepare customised microarray slides in our laboratory. Slides were hybridised with sera from 120 POAG patients and 120 non‐glaucomatous controls (Table 1, microarray validation). With this method, the samples were analysed for their antigen‐autoantibody reactions. Incubation with a fluorescent dye‐labelled anti‐human IgG antibody yielded signal intensities that were proportional to the concentration of the respective autoantibody. After data pre‐processing and normalisation, statistical tests and analysis were conducted. The results of the Mann–Whitney *U*‐test are listed in Table 5. This approach identified significant increased autoantibody levels to PNMA2, TARS, C1QBP and HSPD1 (Figure 7). Signals representing the binding of ACTN1, IQGAP1 and MYO1C AAbs were at the detection limit of this method and therefore could not be quantitatively evaluated.

**Table 5:** Results of microarray validation

	Rank Sum POAG	Rank Sum CTRL	*U*	*Z*	*P*‐value	*Z* adjusted	*P*‐value
PPP2R2B AAbs	14 078	14 842	6818	−0.710	0.477	−0.712	0.477
TARS Aabs	16 741	12 179	4919	4.242	0.000	4.258	< 0.001
C1QBP Aabs	15 714	13 206	5946	2.332	0.020	2.333	0.020
PNMA2 Aabs	16 032	12 888	5628	2.923	0.003	2.930	0.003
MCTS1 Aabs	15 015	13 905	6645	1.032	0.302	1.034	0.301
HSPD1 Aabs	16 793	12 127	4867	4.338	0.000	4.350	< 0.001

Comparison of autoantibody levels in POAG and CTRL samples (*n* = 120) assessed by a two‐sided Mann–Whitney *U*‐test.

### Correlation with disease‐related clinical parameters

We wanted to examine further whether the detected increase in PNMA2, TARS, C1QBP and HSPD1 AAbs showed any relationship to clinical hallmarks of POAG pathogenesis. Calculation of Spearman's rank‐order correlation coefficients was used to detect correlations of the AAbs with cup disc ratio (CDR), visual field defect (‘mean deviation’; MD) and IOP (Table 6). The correlation coefficients reveal a weak negative correlation of PNMA2 and HSPD1 AAbs with CDR (*R* = −0.29 and *R* = −0.28), a moderate negative correlation of HSPD1 AAbs with visual field defect (*R* = −0.42) and a weak positive correlation between TARS AAbs and visual field defect (*R* = 0.34). None of the AAbs showed a relation to IOP. To investigate whether these AAbs show significant alterations in other systemic or eye diseases, we divided the control group into the categories, other eye disease (*n* = 39), systemic disease (*n* = 18), other eye + systemic disease (*n* = 40) and healthy subjects (*n* = 23). Kruskal–Wallis ANOVA for multiple group comparison revealed no significant difference (*P* > 0.05) for PNMA2, TARS, C1QBP and HSPD1 AAbs among these groups.

**Table 6:** Spearman rank correlations of autoantibodies in POAG patients and glaucoma‐related clinical parameters: cup disc ratio (CDR); visual field defects (‘mean deviation’, MD); intraocular pressure (IOP). Spearman correlation coefficients and corresponding *P*‐values.

Clinical parameter	Autoantibody	Valid *N*	Spearman *R*	*t* (*N*‐2)	*P*‐value
CDR	C1QBP	82	0.082	0.732	0.466
	PNMA2	82	−0.286	−2.673	0.009
	TARS	82	0.098	0.884	0.379
	HSPD1	82	−0.284	−2.648	0.010
MD	C1QBP	55	0.113	0.826	0.413
	PNMA2	55	−0.155	−1.140	0.260
	TARS	55	0.335	2.586	0.012
	HSPD1	55	−0.421	−3.376	0.001
IOP	C1QBP	114	−0.120	−1.279	0.203
	PNMA2	114	0.091	0.964	0.337
	TARS	114	0.085	0.905	0.367
	HSPD1	114	0.037	0.396	0.693

### Evaluation of diagnostic potential

**Figure 8:**
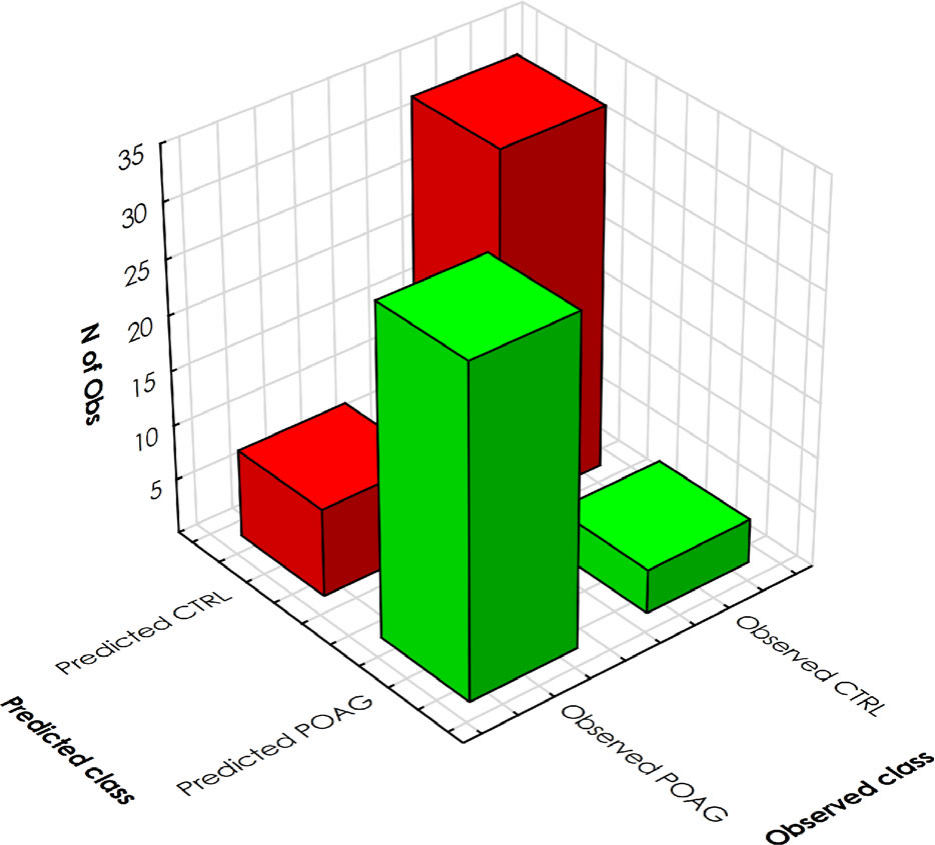
Random forest classification. Prediction is plotted using test samples not used for training (*n* = 75) only. Bars represent the number of predicted (POAG in green; CTRL in red) and observed cases. The random forest algorithm classified POAG samples and CTRL samples with a sensitivity of 79%, a specificity of 89% and 84% accuracy.

The establishment of reliable biomarkers for the objective diagnosis of POAG is still a crucial task in ophthalmic research. Therefore, we wanted to evaluate whether validated glaucoma‐related AAbs to PNMA2, TARS, C1QBP and HSPD1 hold the potential to serve as biomarker candidates. For this purpose, we applied a random forest algorithm to the microarray data set to seek a potential classification of POAG patients and non‐glaucomatous controls. The data set was randomly divided into a training (*n* = 165) and a test set (*n* = 75). The resulting random forest model was able to correctly classify 30 out of 38 POAG patients and 33 out of 37 controls (Figure 8). This translates to a sensitivity of 79% at 89% specificity with an overall accuracy of 84%.

## Discussion

Antibodies to self‐antigens are mostly associated with immune disorders such as systemic lupus erythematosus^28^, ^29^ or Hashimoto's disease,^30^ but are also frequently found in conditions that are not considered autoimmune. Antibodies to tumor antigens are present in the blood of cancer patients,^31^ and AAbs are even found in neurological disorders such as Alzheimer's^32^ or Parkinson's disease.^33^ In recent years, evidence has increased that AAbs are not only involved in pathology, but are also a part of physiologic processes. An important goal for the better understanding of the role of immunological processes in health and disease is to link the natural AAbs to specific biological processes and signalling pathways. Also, the formation of autoantigens in relation to diseases has not been elucidated conclusively. So far, several causes for proteins to become immunogenic have been discussed. These comprise the hypothesis of molecular mimicry,^34^ but alterations in post‐translational modification, protein overexpression, alternative splicing and the generation of neo‐epitopes in the course of apoptosis have also been proposed.^35^, ^36^, ^37^, ^38^ These proposals also infer that differences in circulating autoantibody abundances might reflect molecular changes. However, most identified autoantigens have not been studied for the underlying reason for their immunogenicity. Although the possible involvement of the immune system in glaucoma pathogenesis has been investigated in many studies, the qualitative characterisation of the involved antigens and AAbs is incomplete. The specific conditions under which an antigen can lead to a modified autoimmunity are also unclear. There is a further need to understand the nature and function of the natural autoantibody repertoire as a whole. That will also significantly help to understand the changes that occur under different disease states. In this study, we investigated the autoantibody repertoire against TM proteins as part of natural autoimmunity as well as POAG‐related alterations. This exploratory study also aims to stimulate the formation of hypotheses that would help to explain the properties of the natural immunome and the implications of autoimmunity in glaucoma.

We analysed autoantigens in healthy and glaucomatous TM cells that were captured by serological antibodies of POAG and non‐glaucomatous controls using MS‐AMIDA. At first, we wanted to identify targets of the naturally occurring AAbs and link them to biological processes and signalling pathways. To this end, we performed GO enrichment analysis with the 66 proteins captured by antibodies isolated from non‐glaucomatous sera. The GO analysis involved the evaluation of three different categories, ‘biological process’, ‘molecular function’ and ‘cellular component’. This analysis showed that there is a strong association of the antigens targeted by naturally occurring AAbs with translational processes as part of protein biosynthesis. Interestingly, several enzymes of the family of tRNA ligases were identified as immunogenic proteins. Many proteins representing subunits of the aminoacyl‐tRNA synthetase multienzyme complex were also enriched in different cellular components. However, the AAbs also directly targeted epitopes of the 40S ribosomal subunit. This was also reflected by the strong enrichment of RNA‐binding properties in the identified antigens.

Furthermore, the antigens were highly enriched in the cellular components, ‘membrane’ and ‘cytosol’. They were, however, also located in ‘extracellular exosomes’ and the ‘nucleoplasm’. That the autoantigens are also involved in intranuclear processes was also reflected by their enrichment in poly (A)‐RNA‐binding properties and nuclear‐transcribed mRNA processing in the control mechanism of nonsense‐mediated decay. Another study on the overall immunogenicity of autoantigens could also show a frequent enrichment of autoantigens with a function of RNA and protein binding and also a strong association with ribosomes and spliceosomes.^39^ The abundance of antigens associated with viral transcription‐related processes may be because the cell lines used in this experiment were transformed by simian virus 40^40^ that could be responsible for changes in cellular transcription proteins.

The most abundant antigens identified were nuclear proteins. At 23.3%, histone proteins were the most common, followed by the histone‐binding protein SET. Anti‐histone antibodies have been linked to the autoimmune disease systemic lupus erythematosus,^28^ but our results indicate that they might also be involved in natural autoimmunity. AAbs to ribonucleoproteins are associated with a spectrum of rheumatic diseases.^41^ It is presumed that RNA‐binding proteins can activate B cells and thereby enable AAb production by interaction of the RNA with TLR‐7 or TLR‐8.^42^ Since we have also found these antibodies in individuals who are not suffering from an autoimmune disease (according to anamnesis), there is also the probability that anti‐ribonucleoprotein antibodies are also part of the natural autoantibody repertoire. This also suggests that other criteria would need to be met for these AAbs to become pathogenic.

The second goal of this study was to investigate possible alterations in the immunoproteome in POAG patients. To this end, we compared the binding of serological antibodies, purified from POAG patients and non‐glaucomatous controls, to proteins derived from GTM and HTM cells. The autoantigens, identified and quantified by LFQ, showed more pronounced alterations in the groups with a different protein source (GTM vs HTM) than in the CTRL vs POAG sera groups. Antibody reactivity seems to be more influenced by possibly disease‐related antigen properties than by mere abundance of the antibodies. This could indicate that not only the antibodyome is changed during disease, but that natural AAbs bind with higher affinity to antigens with disease‐related alterations. Our data do not provide further insights into the nature of these alterations, but the occurrence of neo‐epitopes, specific altered post‐translational modifications or truncations are possible changes that could enhance the target binding of physiological AAbs. These explanations need to be evaluated through additional research, so the implications we can make from these results are merely theoretical. A hypothesis that needs further validation by subsequent experiments is that not only quantitative but also qualitative differences between natural and disease‐related AAbs may play a role. Hints that support such assumptions come from other studies that show the disease‐specific accumulation of distinct complementarity determining region peptides (CDR peptides) of the highly variable region of AAbs in glaucoma and other diseases.^43^, ^44^, ^45^ From these findings, it seems likely that not only alterations of the antigen but also of the AAbs themselves can lead to the onset of autoimmune processes. This raises an interesting question for future studies that aim to analyse whether alterations of either antibody or target protein can influence the binding properties. Also, the possible ability of these alterations to promote undesirable effects that may accompany or even accelerate pathological processes needs to be investigated.

The PPI network and pathway enrichment analysis of the POAG‐related autoantigens using Metascape revealed that especially those proteins involved in translation and the PDGFRB pathway show altered immunogenicity. Also, the five connected proteins, HSPD1, CAD, YWHAZ, EIF5A and OLA1, as identified by MCODE, are strongly enriched in these molecular processes. PDGF signalling has been linked to fibrotic diseases and is implicated in the proliferation of cells with myofibroblast‐like properties.^46^ TM cells also show similar characteristics to contractile myofibroblasts that express alpha‐smooth muscle actin (a‐SMA).^47^ Fibrosis also involves an accumulation of ECM, which was also observed in the glaucomatous TM.^48^ The hypothesis of an involvement of fibrotic processes in POAG‐related modulations of TM that could be mediated by increased levels of transforming growth factor β2 (TGFβ2) in the aqueous humour has already been discussed.^49^, ^50^ The MCODE cluster of AAbs potentially reflects POAG‐specific alterations of the PDGFRB pathway. However, a causative or synergetic effect of these AAbs in mediating autoimmune‐driven fibrosis in glaucomatous TM cannot be excluded. Thus, an investigation of the effects of AAbs on TM cells seems to be required. In a previous study from our group, a retinal ganglion cell line was incubated with serum or serological antibodies alone, derived from POAG patients or non‐glaucomatous controls.^51^ In this experiment, no changes in cell viability were observed. However, a change in protein expression towards a pro‐apoptotic state occurred when incubated with POAG serum or isolated POAG IgG, but not after incubation with control serum. We assume that the antibodies would exert similar effects on TM cells. Nonetheless, this would be an interesting subject for further studies, especially as the interaction of autoantibodies with targets in the PDGFR pathway of TM cells could give new insights into their role in glaucoma pathogenesis.

In other studies, which examined autoantibodies in glaucoma, but also in other neurodegenerative diseases such as Alzheimer's or Parkinson's disease, a disease‐specific change in the levels of various serum autoantibodies could be shown. We presume that an altered interplay of antigens and autoantibodies underlies both qualitative and quantitative changes that must be taken into account. Therefore, we wanted to analyse whether the antibody levels to the antigens identified here also show a change in abundance that can be detected by a more sensitive method. To analyse the relative abundance of the serological AAbs to selected targets in glaucomatous and non‐glaucomatous subjects, we used a protein microarray approach as a high‐throughput method. Even though specific attributes of the AAbs and their respective antigens seem to play a significant role in the characteristic of their interaction, many previous studies have also shown that disease‐related alterations of natural autoimmunity can also be related to the levels of the serological AAbs. The evaluation of these quantitative differences is especially valuable in disease diagnostics, as they are easier to measure in clinical tests. We used recombinant human proteins, produced with a wheat germ expression system, generating proteins without post‐translational modifications, to achieve a standardised experiment set‐up. The analysis of sera from 120 POAG and 120 non‐glaucomatous subjects revealed an increase of anti‐C1QBP, anti‐TARS, anti‐PNMA2 and anti‐HSPD1 AAbs in the POAG group. While antibodies to HSPD1 (mitochondrial 60 kDa heat shock protein) have frequently been observed in association with glaucoma,^18^, ^52^, ^53^, ^54^ AAbs to C1QBP, TARS and PNMA2 have not yet been described in the disease context.

Component 1 Q subcomponent‐binding protein (C1QBP) is a multifunctional protein and putative receptor for component 1Q. This function leads to the inhibition of the C1 complex and thereby to suppression of complement activation. It is also involved in the regulation of mRNA splicing via its RNA‐binding capacity. Our results showed upregulated levels of anti‐C1QBP antibodies in the serum of POAG patients. Although AAbs to C1QBP have not yet been described in glaucoma, activation of the complement system has been discovered in the retinal ganglion cells of glaucomatous eyes.^55^ C1QBP AAbs could presumably prevent the inhibition of C1 complex formation by blocking the C1QBP effector sites.

Our study also showed increased levels of autoantibodies to threonyl‐tRNA synthetase (TARS). TARS is an enzyme functioning as the ligase of threonine to its respective tRNA. Neither TARS AAbs nor the antigen itself has been investigated in the context of glaucoma. It cannot be clearly stated what causes the formation of these AAbs and whether they are involved in disease‐related tissue damage. Antibodies to different other tRNA synthetases are often found in patients with inflammatory myositis, especially Jo‐1 AAbs.^56^ Although these myositis‐specific AAbs are far better characterised, researchers did not propose the reason for their occurrence, or the implications for the disease.^57^ The characterisation of the origin and the properties of the TARS AAbs remains a task for further studies.

The paraneoplastic antigen 2 (PNMA2) is known to be linked to paraneoplastic disorders and is also related to neurodegenerative processes. AAbs are frequently found in cancer patients, as tumor cells are suspected of producing abnormal amounts of different members of the PNMA family.^58^ These proteins are also naturally expressed by neuronal cells. The presence of this protein in TM cells is conceivable, since this tissue is likely to have originated from the neural crest.^59^ Also, other PNMA proteins could be identified in porcine TM.^60^ It is assumed that AAbs to PNMA2 are produced as a consequence of protein overexpression in cancer cells. But it cannot be excluded that other properties of the antigen are responsible for its immunogenicity as well. The PNMA2 protein seems to be prone to an autoimmune reaction, which could also be relevant to other neurodegenerative diseases such as glaucoma. However, the AAbs do not seem to drive the progression of paraneoplastic neurologic disease, since their depletion did not result in relief of the symptoms, nor can the injection of the AAbs induce the disease.^61^ It could be an interesting topic for further studies to investigate the specific role of PNMA family members in the pathogenesis of glaucoma.

The identified AAbs seem to be associated with POAG but are mostly not exclusive to glaucoma. This is also true for several glaucoma‐related AAbs previously identified. For example, antibodies to HSP60, vimentin and annexin A5 have not only been reported in association with glaucoma,^18^, ^23^, ^25^, ^52^, ^54^ but are also frequently found in the sera of cancer patients.^62^, ^63^ Also, the antigens are not TM‐specific. Therefore, they might not be specific enough to serve as biomarker candidates. Although the application of random forest algorithms in this study enabled classification of POAG patients with a sensitivity of 79% at 89% specificity, a translation of these finding to be used in diagnostics might not be feasible and therefore needs additional evaluation. As observed with other autoantibody disease markers, variability in AAb levels in the population can be very high. Biomarkers often lack specificity since the putative disease markers are also widespread among subjects without the respective disease.^64^ The establishment of reliable biomarkers requires further knowledge about their roles in glaucoma and their distribution in the general population.

If the occurring autoantibodies are an epiphenomenon of the pathological changes in the TM, alterations in the involved proteins would necessarily occur beforehand. It has been shown that pathways, especially those regulating extracellular matrix deposition, the actin cytoskeleton and cell–cell/cell–matrix connections, are altered in glaucomatous TM. Several factors are thought to play a role in these changes, including increased expression of TGFβ. It seems quite possible that a single occurrence of elevated IOP could lead to proteomic alterations and consequently altered autoantibody profiles. In a previous study, our group was able to highlight changes in autoantibody levels after acute angle‐closure glaucoma attack, an event accompanied by a sudden increase of IOP and probable substantial damage to the optic nerve, in patients without previous glaucoma history.^65^

Although our study provides new insights into the properties and POAG‐related changes of the natural autoantibody repertoire, some limitations shall be pointed out. The proteomic analysis involved in the MS‐AMIDA approach requires a large amount of protein sample material. The availability of human TM tissue samples is limited, and primary cell cultures are also restricted in the amount of material they can yield. For the purpose of our explorative study, we used the most practical TM cell lines. These, however, have the disadvantage of being transformed by simian virus 40 that alters the cellular transcription machinery. Also, the donors of the TM cells were not the same age (GTM cells from a 74‐year‐old POAG patient and HTM cells from an 18‐year‐old donor). This makes further evaluation of our findings using more appropriate TM protein sources mandatory.

## Conclusion

Overall, we could provide new insights into the natural AAb repertoire and highlight pathways which appear to harbour immunogenic proteins. PPI and pathway analysis of POAG‐related autoantigens showed a strong association with the PDGFRB pathway, among others. This pathway is assumed to play an essential role in TM fibrosis, and the related AAbs could play an active role in the pathology or not. Lastly, we could confirm the presence of increased levels of AAbs against PNMA2, TARS and C1QBP in the serum of POAG patients that deserve to be further analysed as potential glaucoma biomarkers.

## Methods

### Sera

The blood samples in this study were collected in accordance with the Declaration of Helsinki on biomedical research involving human subjects. Written informed consent was obtained from each subject. The ethics committee of the Landesärztekammer Rheinland‐Pfalz approved the usage of the samples (Vote: 827.228.11 (7770)). All subjects included in this study received an ophthalmic examination at the Department of Ophthalmology of the University Medical Center in Mainz, Germany. POAG patients were diagnosed according to the guidelines of the European Glaucoma Society,^66^ based on elevated IOP, visual field defects and optic nerve cupping. The control group consists of age‐ and sex‐matched non‐glaucomatous subjects. In the discovery phase using MS‐AMIDA, 30 serum samples of POAG patients and 30 non‐glaucomatous controls were analysed. Subjects with other eye diseases (except cataract), known autoimmune diseases (as evaluated from anamnesis) and previous eye surgeries were excluded to reduce the possible effects of other conditions on the serum antibody levels. With these exclusion criteria, we wanted to exclude bias from immunologic reactions that are not glaucoma‐related or might be a cause of the mechanical disruption of the ocular tissue caused by surgical intervention. This might lead to an exchange of intracellular proteins with blood or aqueous humour that otherwise would not be in contact under physiological or disease‐related conditions and could influence the conclusions drawn from the experiments. In the large‐scale microarray validation, 120 POAG samples were compared to 120 non‐glaucomatous, age‐ and sex‐matched control samples. Here, the exclusion criteria were abandoned, except for other glaucoma types in the control group. The characteristics of the study population can be found in Table 1.

### IgG isolation

Mass spectrometry‐based antibody‐mediated identification of autoantigens was conducted using 30 POAG and 30 control serum samples (Table 2). Samples were pooled, creating three sample pools (10 samples/pool) for each group. IgG was isolated from 2 mL of pooled serum samples by affinity purification, using the NAb Protein G Spin Kit (Thermo Scientific, Rockford, IL, USA), according to the manufacturer's instructions.

### Cell culture

As the source for TM proteins, the immortalised human TM cell line HTM5 and the glaucomatous TM cell line GTM3 were used. Both cell lines were a kind gift of Professor Abbot F. Clark and colleagues [North Texas Eye Research Institute (NTERI), Fort Worth, TX, USA]. The cell lines are derived from human TM as described elsewhere.^40^, ^67^ Cells were grown in DMEM low glucose medium (Sigma‐Aldrich, St. Louis, MO, USA), supplemented with 10% foetal calf serum (Gibco, Life Technologies, Carlsbad, USA), 1% penicillin/streptomycin solution (Sigma‐Aldrich, St. Louis, MO, USA) and 1% 200 mm L‐alanyl‐L‐glutamine (Biochrom, Berlin, Germany). The TM cells were cultured in an incubator at 37°C and 5% CO_2_. Media were changed every 2–3 days. On confluence, cells were collected using cell scrapers. Cell pellets were snap‐frozen in liquid nitrogen and stored at −20°C until further use.

### Cell lysis

Cell pellets were incubated for 30 min on ice with lysis buffer, modified after Alhamdani *et al.* 68 [20 mm HEPES pH7.9, 1 mm MgCl2, 5 mm EDTA, 1 mm PMSF, 0.5% Triton X‐100, 0.5% NP40, 0.25% ASB‐14, 0.25% CHAPS (Carl Roth, Karlsruhe, Germany), 0.5% Protease Inhibitor Cocktail, 0.5% Phosphatase Inhibitor Cocktail (reagents obtained from Sigma‐Aldrich, Steinheim, Germany, unless otherwise stated)] with occasional vortexing. Additionally, cells were mechanically disrupted using an ultrasonic probe. The cells were sonicated in short burst, with occasionally cooling of the samples on ice. After an additional incubation for 15 min on ice, the cells were centrifuged at 20 000 *g* and 4°C. Afterwards, the supernatant was collected, and protein concentration was determined using a BCA assay kit (Thermo Scientific, Frankfurt, Germany).

### Immunoprecipitation

For the immunoprecipitation (IP) of autoantigens from TM lysates, isolated IgG from serum was cross‐linked to magnetic Sepharose beads with Protein G as ligand (Protein G Mag Sepharose; GE Healthcare, Freiburg, Germany). The IP was carried out using a modified protocol, based on instructions from the manufacturer. One hundred microlitres of bead slurry was incubated with 2 mg of isolated IgG for 1 h at 4°C on a rotation incubator. Unbound IgG solution was removed, and beads were washed with TBS [Tris (Carl Roth)‐buffered saline; pH 7.5]. For the chemical cross‐linking of the antibodies, beads were first equilibrated in triethanolamine solution [TEA (Sigma‐Aldrich, Steinheim, Germany); 200 mm; pH 8.9] before incubation with dimethyl pimelimidate dihydrochloride (Sigma‐Aldrich, Steinheim, Germany; 50 mm in 200 mm TEA; pH 8.9) for 30 min with slow rotation at room temperature. Beads were washed with TEA solution following 30‐min incubation in 100 mm ethanolamine (Sigma‐Aldrich, Steinheim, Germany; pH 8.9). The beads were washed once with elution buffer [0.1 m Glycine (AppliChem, Darmstadt, Germany)‐HCl (Carl Roth), 2 m urea (Carl Roth); pH 2.9] and two times with TBS, to reduce unspecific binding. The beads covalently bound to the antibodies were incubated overnight with 2 mg TM protein (1 mg mL^−1^) at 4°C on a rotation incubator. After removing unbound TM proteins, the beads were washed three times with TBS and then four times with 100 mm ammonium bicarbonate solution (ABC; Sigma‐Aldrich, Steinheim, Germany).

### On‐bead digestion

For the preparation of the samples prior to MS analysis, on‐bead tryptic digestion of the precipitated proteins was used, as described in Ref.^69^ To this end, 30 µL of trypsin solution (Promega, Madison, WI, USA; diluted in 100 mm ABC) was directly added to the beads following incubation for 15 min at room temperature with occasional vortexing. After overnight incubation at 37°C, supernatant was collected and stored at 4°C. Another 30 µL of trypsin solution was added to the beads before incubation for additional 4 h at 37°C. The supernatant was separated from the magnetic beads, and both digests were pooled. Formic acid (Merck, Darmstadt, Germany) was added to a final concentration of 5%. The samples were then dried in a vacuum concentrator. Prior to MS analysis, samples were diluted in 0.1% trifluoroacetic acid (TFA; Merck) in HPLC‐grade water (AppliChem) and purified using SOLAµ SPE plates (HRP 2 mg mL^−1^ 96‐well plate; Thermo Scientific, Rockford, IL, USA) using the manufacturer's protocol with slight modifications.^70^ In brief, the plate was activated with 150 µL acetonitrile (ACN; AppliChem) and equilibrated with 0.1% TFA solution. Samples were loaded three consecutive times on the plates, followed by two washing steps with 0.1% TFA. Peptides were eluted twice with 25 µL 60% ACN. All samples were lyophilised by vacuum centrifugation (SpeedVac, Thermo Scientific, Waltham, MA, USA) and stored at −20°C until MS analysis.

### LC‐ESI‐MS/MS

Following on‐bead tryptic digestion, the samples were analysed using an LC‐ESI‐MS/MS system (LTQ Orbitrap XL; Thermo Scientific, Rockford, IL, USA).^60^, ^71^ The samples were first solubilised in 10 µL of 0.1% TFA. The LC system consisted of a 30 × 0.5 mm BioBasic C18 column (Thermo Scientific, Rockford, IL, USA) and a Rheos Allegro pump (Thermo Scientific, Rockford, IL, USA). A PAL HTC autosampler (CTC Analytics, Zwingen, Switzerland) was used to inject 6 µL of the samples into the system, followed by a solvent gradient. The gradient was run for 120 min per sample: 0–40% solvent B (0–40 min), 40–80% solvent B (40–80 min), 80–100% solvent B (80–100 min), 100–80% solvent B (100–110 min), 80% solvent B (110–120 min) (solvent A: LC‐MS‐grade water (AppliChem) + 0.1% (v/v) formic acid; solvent B: LC‐MS grade ACN + 0.1% (v/v) formic acid). The LC system was coupled to an electrospray ionisation (ESI)–LTQ–Orbitrap XL MS (Thermo Scientific, Bremen, Germany) for the acquisition of the mass spectra data. The system was operated in a data‐dependent mode of acquisition to switch between Orbitrap‐MS and LTQ‐MS/MS acquisition automatically. The detection range was set to 300–2000 m/z with a resolution of 30 000. Parameters for dynamic exclusion were set to a repeat count of 1, a repeat duration of 30 s, with an exclusion list size of 50 and exclusion duration of 90 s. Collision‐induced dissociation (CID) fragmentation was used to isolate the five most intense precursor ions for fragmentation in the LTQ. Activation time was set to 30 ms with a repeat count of 10. Normalised collision energy (NCE) was set to 35%.

Mass spectrometry spectra were analysed using MaxQuant (version 1.5.3.30; Max Planck Institute of Biochemistry, Martinsried, Germany). The spectra were searched against UniProt reviewed protein database (*homo sapiens*; 11.02.2019). Mass tolerance was set to ±20 ppm for precursor ions and ±0.5 Da for fragmentation. Carbamidomethylation of cysteine was set as fixed modification, N‐terminal acetylation and oxidation of methionine as variable modifications. Trypsin was chosen as the digestive enzyme, and a maximum of two missed cleavages were allowed. Identification was based on a minimum peptide length of 7 with a FDR < 0.01. Proteins were quantified according to their peptide intensities using MaxQuant label‐free quantification (LFQ).

### Antigen microarray analysis

Selected autoantigen candidates were purchased as recombinant proteins with no post‐translational modifications (wheat germ expression system). All antigens are listed in Supplementary table 4. The arrays were produced in our laboratory using a non‐contact array printer (SciFLEXARRAYER S3, Scienion, Berlin, Germany). The selected antigens were spotted in triplicate onto nitrocellulose‐covered glass slides (AVID Oncyte, 16 Pad NC slides, Grace Bio‐Labs, Bend, OR, USA). A human IgG mix (Sigma‐Aldrich, St. Louis, MO, USA) and PBS (Life Technologies, Paisley, UK) were included as positive and negative control spots. The spotting procedure was carried out in a humidity chamber at 60% humidity. After the array spotting, slides were allowed to dry on the spotter platform overnight. Array hybridisation was performed using 16‐well incubation chambers (ProPlate Multiwell chambers, Grace Bio‐Labs). All incubation steps were carried out on an orbital shaker at 4°C. At first, the arrays were incubated for 1 h with a blocking buffer (Super G, Grace Bio‐Labs) to reduce background signals. Then, the blocking buffer was discarded and the residual buffer was removed by washing the slides with phosphate‐buffered saline containing 0.5% Tween‐20 (PBST; Sigma‐Aldrich, Steinheim, Germany) three times. Afterwards, the arrays were incubated with 100 µL diluted serum samples (1:250 in PBS) overnight. PBS‐only negative controls were included on each slide. Next, slides were again washed three times with PBST followed by incubation with an anti‐human antibody conjugated with a fluorophore (Alexa Fluor^®^ 647 AffiniPure Goat Anti‐Human IgG, Fcγ fragment‐specific, 109‐605‐008, Jackson ImmunoResearch, West Grove, PA, USA) as secondary antibody at a 1:500 dilution in PBS for 1 h. After this step, the arrays were washed twice with PBST and twice with ultrapure water. The slides were subsequently dried for 2 min in a vacuum centrifuge concentrator.

Array images were acquired as 16‐bit TIF files using a high‐resolution confocal laser scanner (428 Array Scanner, Affymetrix, Santa Clara, CA, USA). The image analysis software Imagene (Imagene 5.5, BioDiscovery Inc., Los Angeles, CA, USA) was used to quantify spot intensities. Poor‐quality spots were manually flagged and removed from the analysis.

### Microarray data pre‐procession

Net signal intensities were calculated by subtraction of local background intensity. Signals reaching negative values after background subtraction were treated as missing data. Signals derived from the negative control included on each slide were subtracted from each spot to take unspecific binding of the secondary detection antibody into account. Intensities from the triplicate spots were averaged, resulting in one mean fluorescence intensity. All signals were then normalised to the IgG control spots included on each subarray by median centring to reduce intra‐slide variability and batch effects. Therefore, IgG median signal intensities were divided by the overall IgG signal median to attain a normalisation factor for each subarray. All further analyses are based on these normalised fluorescence intensities (NFI). To ensure robustness of the data set and reduce the influence of outliers, values below the 5th and above the 95th percentile were set as missing data. Targets with more than 25% missing data overall were not included in the statistical analyses. Missing data of targets with < 25% missing values were imputed using the k‐nearest neighbour (KNN) algorithm.

### Statistical analysis

The statistical analysis of the MS data was performed with Perseus (version 1.6.2.3; Max Planck Institute of Biochemistry). Before statistical analysis, proteins were filtered, and potential contaminants, reverse hits and proteins only identified by site were excluded. Also, proteins needed to be identified in all three replicates in at least one group. Proteins that were also identified in any of the negative controls were excluded as well. Missing data have been imputed from normal distribution using the inbuilt algorithm to enable statistical testing. Differences in protein abundance were assessed with the Student's *t*‐test using a permutation‐based FDR of < 0.01 as a threshold for statistical significance to correct for multiple testing. Principal component analysis (PCA) was used to show the influence of the protein source on the data variability. Selected target proteins (based on significant altered levels in HTM and GTM samples) were displayed on a heat map with hierarchical clustering based on Euclidean distance using *z*‐score‐transformed LFQ intensities. For PCA and clustering analysis, missing data were replaced by random values drawn from a normal distribution.

Microarray statistics were calculated using Statistica (Statistica 13, StatSoft, Tulsa, OK, USA). Microarray data were evaluated for normality using the Shapiro–Wilk test. The data did not follow a normal distribution (*P* < 0.05), and so, the non‐parametric Mann–Whitney *U*‐test was used for hypothesis testing. Spearman's rank correlation coefficients were used to evaluate the correlations of autoantibody levels and clinical parameters. Kruskal–Wallis non‐parametric ANOVA was used for multi‐group comparisons. A *P*‐value of < 0.05 was considered statistically significant. A random forest classification algorithm was used to evaluate the diagnostic potential of the identified, disease‐related AAbs. Here, 165 cases were used as the training set and 75 samples as the test set.

### Pathway and enrichment analysis

Gene Ontology enrichment analysis was done using DAVID (http://david.abcc.ncifcrf.gov/home.jsp).^72^, ^73^ GO terms with a *P*‐value < 0.05 after Bonferroni adjustment were considered as significantly enriched. Additionally, DAVID functional annotation clusters were used to characterise putative POAG‐related autoantigens. The analysis was carried out with whole *homo sapiens* proteome as background. Metascape (http://metascape.org)^74^ was used for further pathway and enrichment analysis of identified autoantigens. Also, Metascape's molecular complex detection (MCODE) algorithm was used to detect connected network components.

## Conflict of interest

The authors declare no conflict of interest.
