# Supplementary figures and images for: Autoantigens in the trabecular meshwork and glaucoma‐specific alterations in the natural autoantibody repertoire
Source: Clin Transl Immunology. 2020 Feb 29;9(3):e01101. doi: 10.1002/cti2.1101 (PMC7049230; doi:10.1002/cti2.1101)

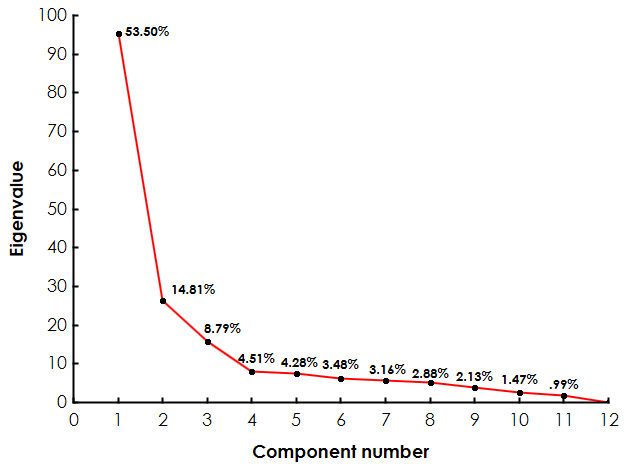

Supplement: Supplementary file 1 [file CTI2-9-e01101-s001.tif]
